# The relationship between the physician and pharmaceutical industry: background ethics and regulation proposals

**DOI:** 10.3325/cmj.2016.57.398

**Published:** 2016-08

**Authors:** Frieder Keller, Krzysztof Marczewski, Draško Pavlović

**Affiliations:** 1 Medical Department I, Nephrology, Ulm University Hospital, Ulm, Germany *frieder.keller@uni-ulm.de*; 2 Department of Nephrology, Endocrinology Hypertension and Internal Medicine, Pope John Paul II Regional Hospital, Zamość, Poland; 3 Sestre Milosrdnice University Hospital, Department of Nephrology and Dialysis, Zagreb, Croatia

Pharmaceutical funding plays an important role in medical progress. Thus, clinical and academic research has been significantly commercialized (1). There are data to suggest that economic interest from industry may have a negative influence on the objectivity of science, research publication, and even patient management (2,3). For example, a manufacturing company has been engaged “… in misleading practices to promote the prescription and usage of rofecoxib, including ‘fake’ journals and guidelines to ‘drug reps’ that minimised the adverse cardiovascular risks” (4). Industry-sponsored reports are up to four times as likely to favor a pharmaceutical company’s product compared to independently published data (5).

For all members of the British National Institute of Clinical Excellence (NICE), influence of industry interest is prohibited. Influence of drug companies can be suspected in 50%-100% of other expert panels as discussed for gabapentin and efalizumab (3,6). Pushing epoetins and cinacalcet, Kidney Disease Outcomes Quality Initiative (KDOQI) guidelines favored target hemoglobin up to 12 mg/dL and recommended calcium values less than 9.5 mg/dL (<2.38 mmol/L) against some evidence (7). As a consequence, it has been suggested that the dynamics of this process needs to be more restricted and governed by less tolerant regulation (8). Considerations of how ethics could be made effective here might help already at an earlier stage.

## Analysis of the present state

The percentage of physicians with any relationship to industry is at 80% or even more (3,9). The primary reason for sponsorship by industry may be subtle psychological effects and expectations of reciprocity (10). Research into the psychology of receiving and giving gifts indicates that more appropriate regulations would be necessary (2,3). While 61% of physicians believed that financial incentive did not influence their own practice, only 16% believed that the same was true for their colleagues (11).

Physicians and scientists do not willingly talk about their own motivations – be they economic or intellectual (10,12). The rigor of a study has been judged to be significantly reduced in studies funded by industry compared to those funded by a government agency (3,13,14).

In response, it has become a prerequisite that all persons involved in the activities of the European Renal Association/European Dialysis and Transplant Association (ERA-EDTA) adhere to the 2014 Council Regulations that were initiated by the ERA-EDTA Ethics Committee (see Acknowledgments). The disclosure – or less adversarially – the declaration of interest is mandatory to make transparent whether there could be a conflict of interest. Such regulations are needed but probably are not sufficient to ensure that conflicting interests are declared and not concealed (15). Research on the tension between moral rules shows “that collaborative settings provide fertile ground for the emergence of corruption” (16).

Regulations will only be instrumental when clear sanctions are implemented (17). Threat of scientific banning, ostracism, litigation, and laws intend to discourage concealment and deception. The German parliament, for example, is planning to issue a new anti-corruption law for workers in the health system (Strafgesetzbuch StGB §299a and b). The boundaries between an illegal incentive that stimulates corruption and financial reward judged to be adequate compensation are fluid. While misuse and non-adherence call for legal regulations or even sanctions, trustworthy actions have a foundation in ethics.

In medicine generally, “the primary interests are the health of the patient,” whereas financial gain, prestige, or preferences are not illegitimate but secondary interests (18). To give an example, financial incentives will motivate adequate measures to reduce mortality and facilitate access to dialysis for all patients who need it. But such economic interest could also corrupt the physician to prematurely recruit patients for dialysis or unnecessarily maintain this treatment (19). According to the medical ethics charter, professionalism “… demands placing the interests of patients above those of the physician” (20). Primary patient interests are welfare, and respect for autonomy and justice (20). Economic mechanisms benefitting the market need special regulations in medicine in which the person who decides, benefits, and pays are not one and the same.

Transparency might help better than potential threats of sanction for non-adherence to the canonical conflict of interest regulations. A researcher could be excluded from receiving any further legal sponsoring, be it from industry or from government. But academic institutions dislike inflicting this ultimate and most efficient sanctioning instrument since this harms the institution itself.

Transparency will help bring the scientific as well as social public to a position from which they are able to judge whether the physician’s interest dominates over the patients’ benefit. Fair payment compensates for work performed, while inadequate payment tends to influence decisions with costly consequences. As professionals, all physicians must seek to gain patients’ confidence, social trust, and vocational reputation (3). In the patient-physician relationship, ethics is the foundation of confidence, and confidence is the foundation of sustainability.

## Proposals

With the most evident objectives in mind, we suggest four proposals on how to deal with a potential conflicting interest when presenting a talk, publishing a paper, or planning a trial (Table 1).

**Table 1:** Proposals for the declaration of interest and how to improve transparency whether a conflict of interest might exist

Targets	The economic interests of clinicians and researchers need to be more transparent
	• Establish sustainable trust and confidence in clinical science, medical practice, and data published from physicians and scientists • Enable patients’, audiences’, or readers’ own judgments as to whether a conflict of interest impacts science or practice

Proposals	How to improve transparency
	1. The conflict of interest should be declared a. on the first slide of any oral presentation and b. on the last line of the abstract as in all New England Journal of Medicine papers (3) 2. For the sake of confidence, the dominant conflict of interest should be declared first (1). 3. Consider mentioning funding by industry also in the patient information form of any investigator-initiated trial. 4. Avoid employees of a company influencing the conduct of a trial and the presentation of data (27,28). It should be part of the primary contract that all contributions by employees or representatives of industry can only be mentioned in the acknowledgment (29).

### Declaration of Conflicts of Interest (CoI)

The declaration of interests does not necessarily mean that the interests are in conflict with truth (3). Conversely, also a declared conflict of interest can still negatively influence science and practice (21). In scientific journals, transparency is needed not only with regard to financial but also intellectual interests (22). The readers and the public must have the opportunity to form their own opinions about the independence and the value of a study (23). An unstructured and unweighted list of many sponsors, although unintentionally (24), could make a contributor falsely appear to the public to be both prestigious and independent (3,14).

To be informed, not only readers and listeners but also patients need complete transparency (25). It should be considered that the amount of financial funding by the industry must be stated for each included subject (at least for the complete study) in the patient information form of any investigator-initiated trial (Table 1). The disclosure is needed whether the money goes to the institution or into the pockets of the investigator. Mistrust will spread when sustainability is neglected. A damaged reputation ultimately results from growing mistrust, as has been discussed with the examples of rofecoxib or the gene therapy for ornithine transcarbamylase deficiency (3,4).

### Authorship

When publishing purely industry-driven research, the name of a highly recognized scientist can be misrepresented as an author. Ghost, guest, or gift authors might make a paper look like good science (15). Another great scientific problem is posed when individuals participate in research, data analysis, and/or writing of a manuscript but are not named or disclosed in the author by-line or acknowledgments (26). If included in the list as co-authors, however, employees or experts acting in charge of pharmaceutical companies can influence the results. As discussed for epoetin or for some psychopharmacological trials, drug company employees could significantly guide the presentation of data (27,28).

Employees can publish their own papers but should not be made co-authors of investigator-initiated trials. This must be clarified by contract from the beginning of the cooperation (Table 1). The contribution to a study by employees or representatives of the industry should exclusively but explicitly be mentioned in the acknowledgments (29).

## Conclusion

Material goods such as medicinal products or medicines can best be manufactured and distributed by market mechanisms; but social and personal relations should still be regulated by moral values (30). True science must not be free from any interest, be it economic or emancipatory (31). In order, however, to maintain academic integrity and to comply with the fiduciary duty of the medical profession, it is in the interest of the credibility of each scientist and every physician to check for possible conflicts of interest.

Intrinsic virtue of professionals should be encouraged and affirmed (3). Physicians will be motivated to keep their own interests under better control by the need to make their interests transparent. A disappointing response to the conflicting interests has been identified as “moral disengagement operations” such as justification, euphemistic labeling, diffusion of responsibility, sharing blame, minimizing risks, and victim dehumanization (32). When patients can no longer trust medicine, controllers and lawyers will dominate the scene (33). Transparency consequently allows for more tolerant regulation because the ethical principles are proactive and not restrictive. On the patients’ side, financial transparency might also bring some illusionary hopes and wishes back to reality.
